# Alcohol and violent deaths in the United States, 2015–2022

**DOI:** 10.1186/s40621-026-00675-4

**Published:** 2026-03-26

**Authors:** Jack Kappelman, Diana Silver, James Macinko

**Affiliations:** 1 UCLA Department of Political Science, Los Angeles, CA USA; 2 Public Health Policy and Management, NYU School of Global Public Health, New York, NY USA; 3 Community Health Sciences, Health Policy and Management, UCLA Fielding School of Public Health, Los Angeles, CA USA

**Keywords:** Firearm, Alcohol, National Violent Death Reporting System, Violent death, Alcohol-involved mortality

## Abstract

**Objective:**

This study describes alcohol involvement in violent deaths using the National Violent Death Reporting System (NVDRS) from 2015 to 2022 in 24 states where at least 90% of decedent records had available alcohol assessment data.

**Methods:**

Using a repeated cross-sectional design, we construct a composite alcohol-involvement measure from toxicology results and NVDRS coder assessments and estimate negative binomial regression models with state-year demographic population counts as offsets to evaluate the roles of alcohol, firearms, and their intersection in violent deaths. The analytic sample included 204,739 decedents.

**Results:**

Alcohol was present in roughly 27% of all violent deaths (27% in suicide and 25% in homicide deaths), with age- and sex-adjusted alcohol-involved mortality rates ranging from 1.6 per 100,000 in Virginia to 7.8 per 100,000 in North Dakota. Firearms and alcohol co-occurred in 19% of homicide and 14% of suicide deaths. Alcohol involvement was most common among men and younger adults (aged 20–39) with the highest rates observed for men aged 30–39 who died from suicide. There was a slight (6%) decrease in mean alcohol-involved violent death rates within a subsample of 18 states observed from 2016 to 2022, although Alaska saw a larger decrease (39%) and Oregon and Connecticut saw a near doubling of their alcohol-involved violent death rates over this period.

**Conclusions:**

These findings indicate that alcohol plays a substantial and complex role in violent deaths. This role differs between homicides and suicides, and varies by sex, age, state, year, and firearm involvement. Violence reduction efforts may benefit from interrupting the links between alcohol consumption and violent behaviors. Improved data collection and toxicology standardization across states would greatly enhance efforts to monitor and evaluate ongoing and future interventions.

**Supplementary Information:**

The online version contains supplementary material available at 10.1186/s40621-026-00675-4.

## Introduction

Violence is a major source of preventable morbidity and mortality in the United States, with firearm-related violence accounting for one of the five leading causes of death among people aged 1 to 44 [1]. In 2022, 48,117 people died from gun-related injuries, a 21% increase from 2019 [2]. While considerable evidence links alcohol consumption to various forms of violence, including homicide, suicide, and unintentional deaths [3], there is a need to better characterize which populations are most at risk when alcohol is involved in a violent death.

Numerous studies have documented the strong association between excessive alcohol use and violence, on outcomes ranging from intimate partner violence, to assault, to suicide [4–11]. Several studies have found alcohol use to be significantly associated with firearm access [12], with firearms as a means of suicide [3], and with unintentional firearm-related deaths [13]. A recent study calculated the alcohol attributable fraction (AAF) for suicide using the 2021 NVDRS, finding that about 1 in 5 suicides involved alcohol, and that the AAF was higher among those using a firearm as compared to those who used other means [14].

Firearms are especially important contributors to violent death and are used in about half of suicide cases in the U.S [15]., and suicide attempts that involve a firearm have higher fatality rates than other means [4]. The role of environmental factors is also well-documented: studies suggest a strong link between firearm-related suicide and firearm prevalence [16], and differences in firearm-involved suicide rates across states are primarily explained by firearm access, rather than other suicide attempts, or individual-level factors such as antidepressant medication usage or the mental health status of the decedent [17–21].

Other studies have examined the relationship between alcohol and firearms on some aspects of violent death. Recent studies using NVDRS data have further explored this intersection, finding dose-response relationships between alcohol and firearm suicide [22], demographic variations in this association [23], and a sharp increase in alcohol-involved firearm suicide at the minimum legal drinking age [24].

While the literature points to an important role of alcohol, less is known about the extent to which alcohol is involved in violent deaths across states, how this relationship may have changed over time, or the ways in which alcohol consumption and other individual-level factors, including firearm usage, may co-occur among decedents and vary across demographic groups. This study evaluates these relationships through an analysis of the National Violent Death Reporting System (NVDRS).

## Methods

### Data

This study uses the Restricted Access Database (RAD) of the NVDRS, 2015–2022. The dataset links data from vital records, coroner/medical examiners’ reports, and law enforcement agencies for participating states to create the most comprehensive and detailed data available on violent deaths. However, not all states participate in every year. The timeline of state participation in the NVDRS can be seen in Table 1.

**Table 1:** NVDRS Participation Across States Over Time, and Variations in Alcohol & Firearm Involvement

Year	(New) States Participating	Num. of Decedents (Full NVDRS)	% Alcohol Involvement (Full NVDRS)	% Alcohol Involvement (Included in Study)	% Firearm Involvement (Full NVDRS)	% Firearm Involvement (Included in Study)
2015	**AK**, **AZ**, **CO**, **CT**, GA, HI, KS, KY, **MA**, **MD**, **ME**, **MI**, **MN**, **NC**, NH, NJ, NM, NY, **OH**, **OK**, **OR**, **RI**, SC, **UT**, **VA**, **VT**, WI	31,593	25.24	27.84	50.11	50.16
2016	IL, IN, **IA**, PA, WA	41,511	23.51	27.23	51.37	50.70
2017	CA, **DE**, DC, **NV**, PR, WV (-HI)	47,463	23.95	27.62	51.19	50.55
2018	AL, **LA**, MO, NE (-HI)	55,225	23.48	27.38	51.08	51.31
2019	HI, MT, **ND**, WY (-NY)	52,593	23.91	27.43	53.15	51.30
2020	AR, **ID**, MS, SD, **TN**, TX, NY (-HI)	66,778	21.19	25.01	56.48	56.17
2021	(-HI)	71,605	20.05	25.74	59.28	58.26
2022	HI, FL	74,959	19.45	25.47	57.32	58.45
	Total:	646,790	22.87	26.58	52.95	53.83

States in bold are those included in our study sample. New states are recorded as such if they did not report to NVDRS in the prior year. If a state ended participation or did not meet participation criteria for a particular year, it is recorded as “(-HI)”, for instance, and rerecorded as a new state if it resumed participation. CA reported data from 4 counties in 2017, 21 in 2018, 30 in 2019, 35 in 2020, 31 in 2021, and 32 in 2022. TX reported data from 4 counties in 2020 and 13 in 2021 and 2022. Hawaii was excluded due to incomplete case recording in 2017, 2018, 2020, and 2021 (though reporting for counties with > 70% of FL’s population was achieved in 2022), as was NY in 2019 and Florida in 2020, 2021, and 2022. From 2016-19, PA and IL collected data on 80% of violent deaths in the state, as did WA in 2016-17. Source: Availability of VDRS Program Data for NVDRS Web-based Injury Statistics Query and Reporting System and Restricted Access Database

We utilize the NVDRS data to construct four samples: (1) decedents of any violent death; (2) decedents of deaths due to homicide; (3) decedents of deaths due to suicide; and (4) decedents of deaths due to causes other than homicide or suicide. Of the 204,739 decedents in our entire sample, 61% died by suicide, 25% died by homicide, and 14% died by other causes (undetermined intent is the largest category totaling 12% of all other causes). We excluded a small number of observations with missing data on sex (*n* = 19), race/ethnicity (*n* = 1,140), age (*n* = 17), and firearm involvement (*n* = 1,816), representing less than 1.5% of the full analytic sample.

### Measures

#### Alcohol and Firearm Involvement

We constructed a composite measure of alcohol involvement to capture cases where alcohol use was either confirmed through toxicology testing or suspected by NVDRS coders. The NVDRS variable for alcohol suspicion is coded as 1 (“Yes”) if there is scene-based evidence from the decedent narrative(s) that the victim used alcohol in the hours before the incident, 0 (“No”) if there is no such evidence, and “not applicable” or “unknown” when the question does not apply or no information is available to assess alcohol use (with toxicology results explicitly excluded from this determination). Using this measure, we then coded alcohol involvement as present if a toxicology test was positive, regardless of suspicion status, or if alcohol use was suspected (by trained NVDRS coders) but no toxicology result was available. Conversely, alcohol involvement was coded as not present if a toxicology test was negative or if alcohol use was not suspected in the cases where no toxicology result was available. Neither measure was present in 7,972 (3.7%) of cases and these were dropped from analysis.

When assessing the accuracy of the NVRDS-coded alcohol suspicion measure, in the subset of 138,453 cases where both alcohol suspicion measures and toxicology testing were available, we calculated sensitivity at 52% and specificity at 95%. This suggests that our approach (relying on toxicology results when present and using the suspicion measure only when toxicology results are not available) is conservative and unlikely to overcount alcohol involvement. Further details on toxicology testing are presented for each state in Appendix Fig. 1. The completeness of these underlying measures across the entire selected NVDRS sample from 2015 to 2022 can be seen in Appendix Fig. 2.

**Fig. 1:**
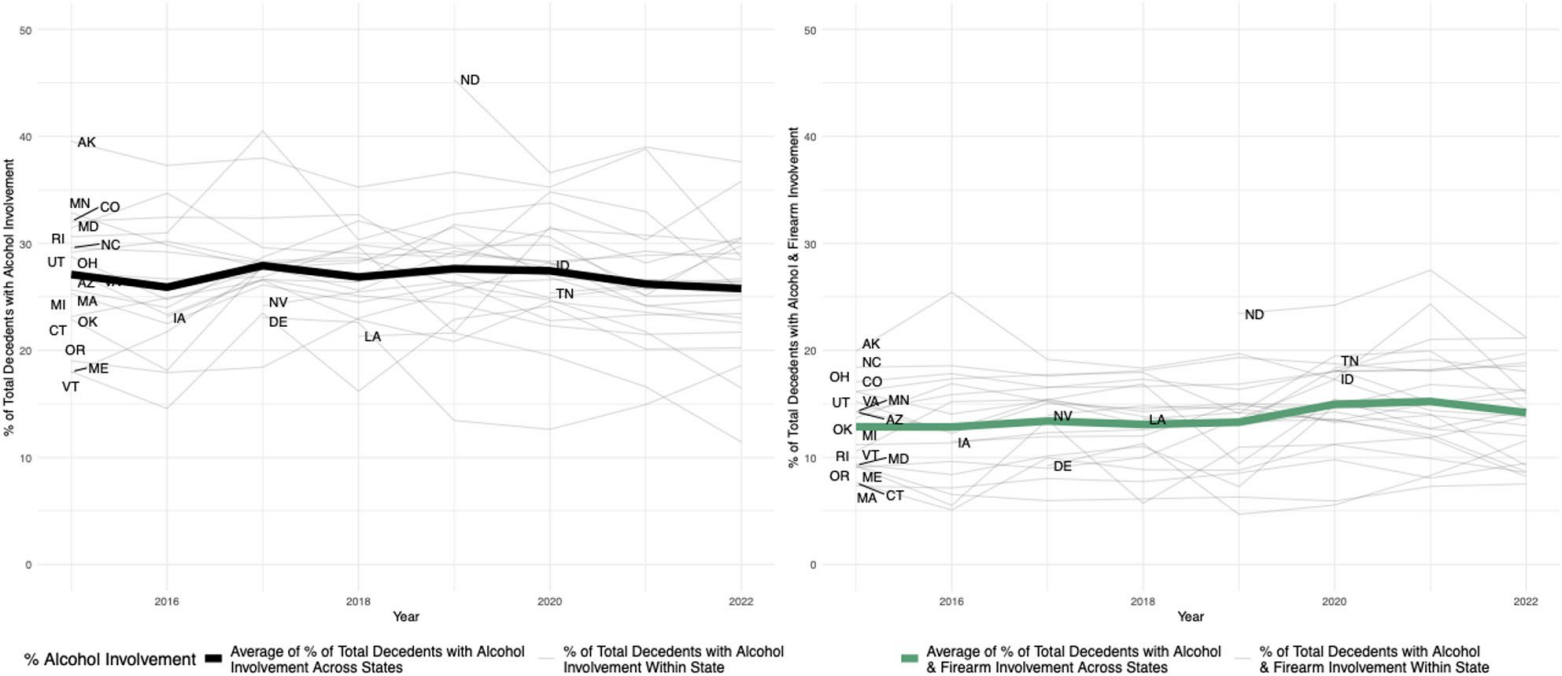
Percent Alcohol Involvement (left) and Alcohol and Firearm Involvement (right) in All Violent Deaths by State and Year, 2015–2022. The left panel shows the percentage of all decedents in the study sample whose deaths involved alcohol, with individual state-level percentages plotted annually and the overall average across states represented by the black line. The right panel focuses exclusively on deaths that involved both alcohol and a firearm, with individual state-level percentages as lighter color lines and the average across states in green. State abbreviations mark the year in which they began participation in the NVDRS

**Fig. 2:**
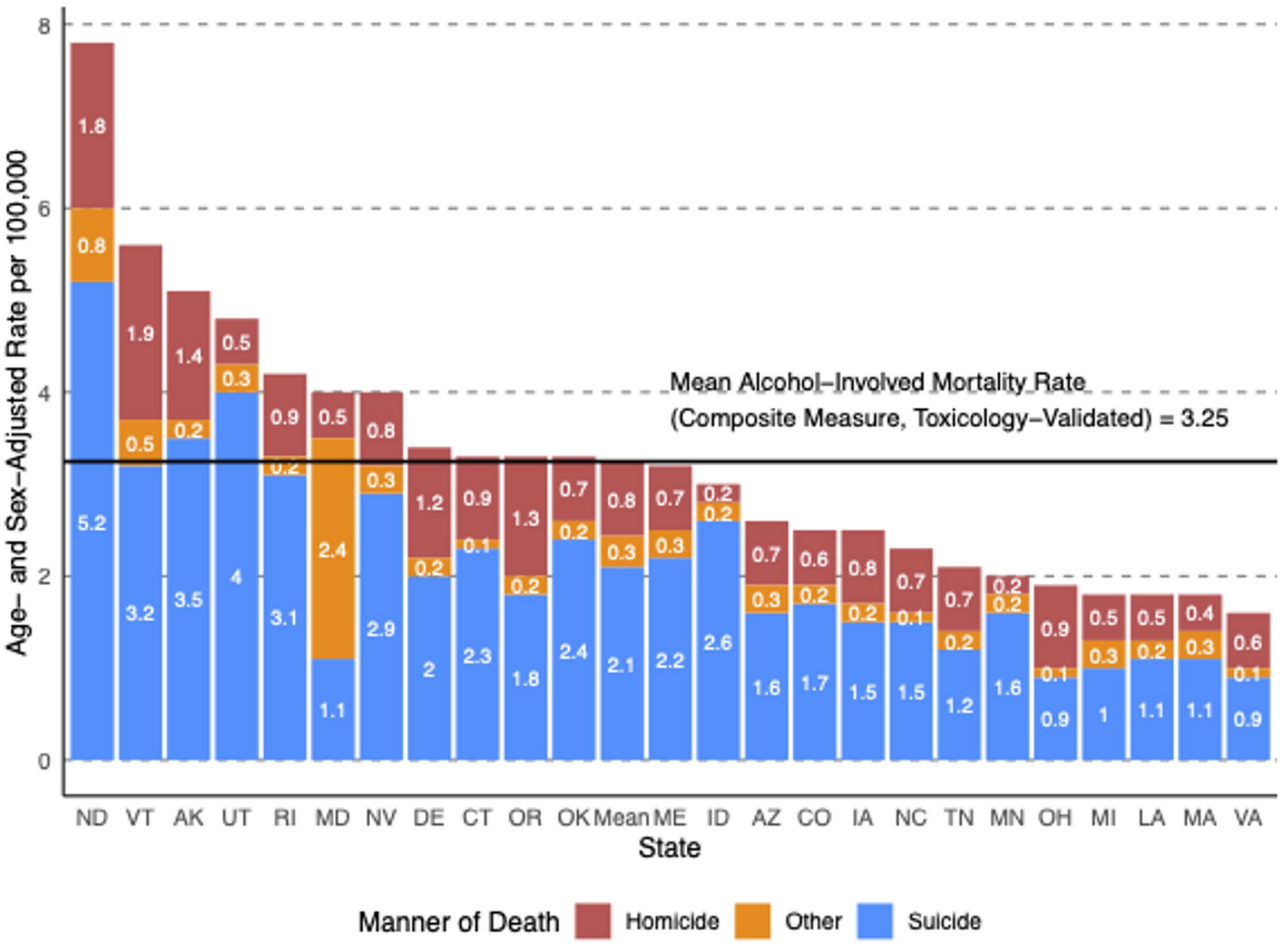
Adjusted Alcohol-Involved Mortality Rate by Manner of Death, 2022. This figure shows the age- and sex-adjusted alcohol-involved mortality rates per 100,000 people by state and manner of violent death (homicide, suicide, and other causes) for 2022. Bars are stacked to represent the total alcohol-involved mortality rate for each state, with component rates labeled within each segment. States are ordered by total rate in descending order. The horizontal line marks the 24-state average total rate (3.25 per 100,000)

Firearm involvement is determined using NVDRS variables that specify the weapon responsible for the fatal injury when a weapon was involved. NVDRS staff manually code these variables for each decedent. See Appendix Fig. 3 for more detail on this measure. We classify a death as involving a firearm if a firearm is listed as the primary fatal weapon.

**Fig. 3:**
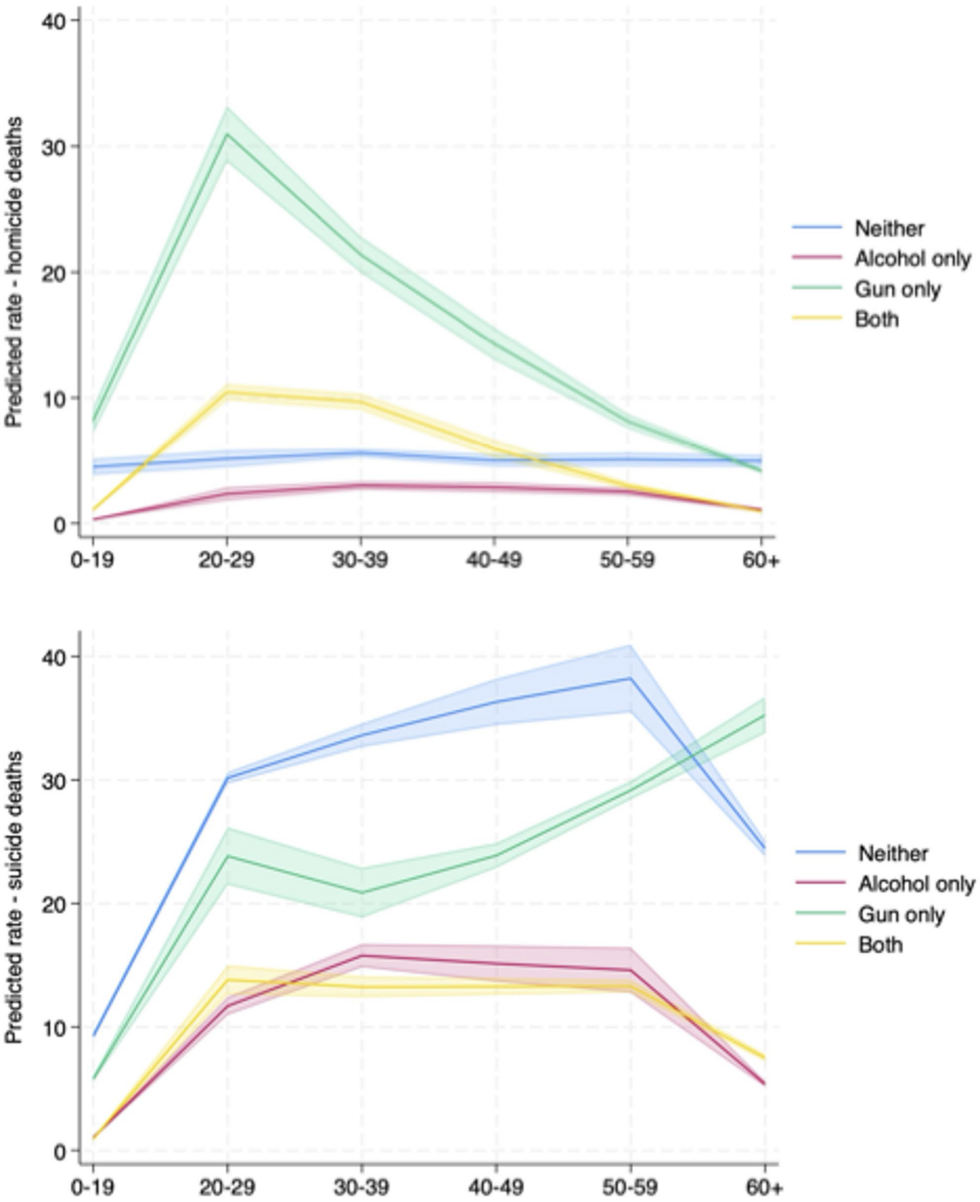
Alcohol and Firearm Involvement in Predicted Homicide (top) and Suicide (bottom) rates, by Age and Sex, 2022. Predicted rates and 95% confidence intervals from negative binomial regression of each outcome controlling for age, sex, alcohol and firearm involvement, state, year, age x alcohol/firearm interactions and log state population as an offset. Data source: NVDRS 2015–2022, for 24 states with over 90% of decedents having recorded alcohol involvement measures

Based on these measures, we then classified all deaths as involving (1) alcohol (without firearms); (2) firearms (without alcohol); (3) both alcohol and firearms; and (4) neither alcohol nor firearms. Our final sample includes all states that participated in the NVDRS over the available time period and in which at least 90% of all deaths had at least one of the alcohol measures present during the study period. A map of included states is presented in Appendix Fig. 4.

#### Individual-level factors

Individual-level covariates include sex, age group (0–19, 20–29, 30–39, 40–49, 50–59, and 60+), and race/ethnicity (non-Hispanic white, non-Hispanic Black, Hispanic, of any race, and other races). Race/ethnicity was included due to documented differences in violent death risk, with some groups collapsed for sample size considerations.

### Statistical analysis

Because states began reporting to the NVDRS at different times during the study period, our panel is unbalanced. We address this data structure by incorporating state-specific yearly populations into the analysis so that, as states enter the panel, their denominators (the log of the age-, sex-, and race/ethnicity-specific population counts) are included. We calculated descriptive statistics for the entire sample and sub-samples, then constructed regression models for each of our dependent variables. Negative binomial regression models are used instead of Poisson models due to the presence of overdispersion [25], and the log of state-year age-sex- and race/ethnicity-specific populations (from the US Census Bureau) are used as an offset [26]. Including the population structure in the regression analyses allows for an examination of contribution of each explanatory variable standardized for different yearly state population and demographic structures, especially important given that age, sex, and race/ethnicity each have different associations with violent deaths and differ across states.

For each model, we include individual-level factors as well as state-fixed effects to account for unobserved time-invariant geographic factors. Year-fixed effects control for temporal variations and to capture unobserved patterns that may influence the outcomes of interest across different years. We report adjusted incident rate ratios (IRR) from the negative binomial regression estimates and the associated 95% confidence intervals, with robust standard errors. Exponentiated coefficients from these models can be interpreted as the relative contribution of each outcome associated with that variable and assess how these factors differ among states. Results are then used to calculate and graph fully adjusted predicted counts for each outcome.

Finally, in order to compare state rates to one another and over time, we perform direct standardization to the US standard population by age, sex and race/ethnicity [27]. All analyses were performed using Stata 19.5 or R version 4.3.1.

## Results

Data for 204,739 decedents were available for our analyses, representing 95.2% of all decedents from 2015 to 2022 from 24 states that encompass every Census geographic region, with the exception of the Middle Atlantic (see Table 1). Table 1 also shows that among all decedents in our 24-state sample, 26.6% of deaths involved alcohol and 53% involved a gun. There was little difference in firearm involvement between our analytic sample and the entire NVDRS database, but alcohol involvement was 3.7% higher in our sample, which is composed of states where 90% or more of decedents were assessed for alcohol involvement.

In Table 2, decedents were on average 43 years old (SD = 18.7; range: 0 to 103), 62% of decedents were male, and 47% of the sample was White, 22% Black, 18% Hispanic, and 14% other races. Regarding manner of death, 61% were due to suicide, 25% homicide, and 14% due to other causes. Alcohol involvement was similar across all causes of death at approximately 27% of suicide, 25% of homicide, and 29% of other deaths in the sample. Involvement of both alcohol and firearms was less common and occurred in 14%, 19%, and 5% of suicides, homicides, and other deaths, respectively.

**Table 2:** Descriptive statistics of the final sample, 2015–2022

Decedent characteristics	All violent deaths	Any Alcohol involvement	Alcohol (without firearms)	Firearms (without alcohol)	Both alcohol & firearms	Neither alcohol nor firearms
Age (mean)	43.0	41.9	43.2	44	40.7	42.8
Female (%)	37.5	33.3	37.9	36.6	28.4	43.8
Male (%)	62.5	66.7	62.1	63.4	71.6	56.2
White (%)	46.6	52.0	52.8	42.6	51.1	42.8
Black (%)	21.8	20.4	17.7	25.2	23.3	20.7
Hispanic (%)	17.9	16.5	16.5	18.9	16.5	19.9
All other races (%)	13.7	11.1	12.9	13.4	9.1	17.4
Cause of Death						
Suicide (N)	125,300	33,455	15,537	47,739	17,918	43,980
% of all suicide	61.2	26.7	12.4	38.1	14.3	35.1
Homicide (N)	51,389	12,847	3,083	30,114	9,764	8,428
% of all homicide	25.1	25.0	6.0	58.6	19.0	16.4
Others (N)	28,049	8,078	6,788	3,394	1,290	16,577
% of all other	13.7	28.8	24.2	12.1	4.6	59.1
Total (N)	204,739	54,461	25,388	81,281	28,868	68,997
% of Total	100	26.6	12.4	39.7	14.1	33.7

Data source: NVDRS 2015–2022, for 24 states with over 90% of decedents having recorded alcohol involvement measures

In the first column only, percentages are for all violent deaths. The remaining percentages in the table are by row, showing the contribution to each specific manner of death

Figure 1 (left panel) shows annual state-level percentages of violent deaths involving alcohol. While state trends are heterogeneous, the national average remained stable near 27% from 2015 to 2019 before declining slightly through 2022. The right panel shows deaths involving both alcohol and a firearm, accounting for 14% of all violent deaths (19% of homicide deaths, and 14% of suicide deaths). This measure also varied across states, from 6.8% in Maryland to 24.0% in North Dakota. Year-to-year variation within states is more pronounced in the right panel.

Table 3 presents incidence rate ratios (IRRs) from negative binomial regression models. Key patterns include: alcohol involvement was less prevalent than the reference category (neither alcohol not firearms) across all death types; firearm involvement alone was highly prevalent in homicides (IRR = 2.50); and the combination of alcohol and firearms was relatively more common in homicides. Men and younger age groups had higher IRRs for homicide, while older age groups had higher IRRs for suicide.

**Table 3:** Negative Binomial Regression Estimates of Alcohol and Firearm Involvement on Violent Deaths, by Outcome

	All Deaths	Homicide	Suicide	Others
Alcohol (without firearms)	0.49***	0.53***	0.44***	0.49***
*versus neither*	0.46,0.51	0.50,0.56	0.43,0.46	0.46,0.52
Firearms (without alcohol)	1.14***	2.5***	0.93***	0.39***
*versus neither*	1.06,1.23	2.40,2.61	0.90,0.96	0.37,0.42
Both alcohol and firearms	0.55***	1.17***	0.45***	0.18***
*versus neither*	0.51,0.58	1.11,1.23	0.44,0.47	0.17,0.20
Female	0.43***	0.53***	0.4***	0.48***
*versus Male*	0.42,0.44	0.51,0.55	0.39,0.41	0.46,0.50
20–29 years	3.31***	2.41***	3.81***	2.62***
*versus 0–19 years*	3.22,3.40	2.27,2.55	3.65,3.98	2.39,2.88
30–39	3.35***	2.38***	3.68***	3.32***
*versus 0–19 years*	3.29,3.42	2.25,2.52	3.53,3.84	3.04,3.63
40–49	3.28***	2.08***	3.87***	3.16***
*versus 0–19 years*	3.17,3.38	1.97,2.21	3.70,4.04	2.89,3.46
50–59	3.08***	1.61***	3.87***	2.8***
*versus 0–19 years*	2.92,3.24	1.51,1.71	3.70,4.04	2.56,3.07
60+	2.01***	1.06	2.81***	1.37***
*versus 0–19 years*	1.91,2.12	0.99,1.13	2.67,2.96	1.25,1.50
NH Black	2.33***	10.58***	0.75***	2.37***
*versus NH White*	2.20,2.47	10.16,11.02	0.72,0.78	2.22,2.52
Hispanic	1.24***	3.42***	0.87***	1.27***
*versus NH White*	1.19,1.30	3.27,3.59	0.84,0.90	1.18,1.37
All other NH races	1.56***	3.06***	1.36***	1.28***
*versus NH White*	1.51,1.61	2.88,3.26	1.32,1.41	1.17,1.40
2016	1.01***	0.98	0.99	1.08
*versus 2015*	1.01,1.01	0.90,1.05	0.94,1.05	0.98,1.19
2017	1.04***	1.02	1.04	1.02
*versus 2015*	1.04,1.05	0.95,1.10	0.99,1.10	0.93,1.13
2018	1.00	0.88***	1.04	1.01
*versus 2015*	0.99,1.00	0.82,0.95	0.98,1.10	0.92,1.12
2019	0.94***	0.85***	0.97	0.87**
*versus 2015*	0.94,0.95	0.79,0.92	0.92,1.03	0.79,0.97
2020	1.04***	1.05	1	1.04
*versus 2015*	1.04,1.05	0.97,1.13	0.95,1.06	0.94,1.14
2021	1.1***	1.11**	1.07**	1.12*
*versus 2015*	1.09,1.12	1.03,1.19	1.02,1.13	1.02,1.24
2022	1.08***	1.10**	1.08**	1.03
*versus 2015*	1.07,1.09	1.02,1.18	1.02,1.14	0.93,1.13
Lnalpha	0.63***	0.99	0.72***	1.63***
	0.61,0.66	0.96,1.03	0.70,0.73	1.56,1.70

* *p* < 0.05; ** *p* < 0.01; *** *p* < 0.001. Models additionally control for state fixed effects (not shown). Lnalpha presents test for overdispersion

Data source: NVDRS 2015–2022, for 24 states with over 90% of decedents having recorded alcohol involvement measures

The highest IRR by age is among those 20–29 and 30–39 for all deaths and for homicides, while higher IRRs were seen for those aged 40 and over for suicide deaths. Compared to non-Hispanic white populations, non-Hispanic black decedents had the highest IRR for all deaths and for homicides, but had IRRs 25% lower for suicide than did white populations. Hispanic populations had an elevated IRR for all deaths and homicide and lower IRRs for suicide. The group composed of all other races had higher IRRs than the reference population in all areas. Coefficients for each year show that in comparison to 2015, all violent deaths increased over time. Homicides rose in 2021 and stayed elevated until the end of the period. Suicide deaths also increased over time, while the temporal pattern for other deaths was mixed.

Figure 2 illustrates the age- sex- and race/ethnicity-adjusted alcohol-involved mortality rates (per 100,000) for each state in 2022. Each bar represents the sum of that state’s alcohol-involved mortality rate composed of suicide, homicide and other means. The average rate for all states was 3.2/100,000. North Dakota had more than double the average rate at 7.8/100,000. Vermont, Alaska, Utah, Rhode Island, Maryland and Nevada all has rates above the average, while Ohio, Michigan, Louisianna, Massachusetts, and Virginia had rates below the mean. In all states, except Maryland, deaths due to suicide represent the largest component, followed by homicide and other or undefined means.

Table 4 reports changes in the adjusted alcohol-involved violent death rate for the limited number of states that participated in all NVDRS years between 2016 and 2022. The majority of states had little change in rates of alcohol-involved violent death in each period with an average decrease of 0.2/100,000 or 6%. Alaska saw the largest decrease over time (39%), while Oregon, Connecticut, and Maine experienced the largest percent increases at 94%, 83%, and 68%, respectively.

**Table 4:** Differences in age- sex- and race/ethnicity-adjusted alcohol-involved violent death rates per 100,000 by state between 2016 and 2022

State	2016	2022	Absolute Difference	% change
AK	8.3	5.1	-3.2	-38.6%
AZ	2.2	2.6	0.4	18.2%
CO	2.5	2.5	0	0.0%
CT	1.8	3.3	1.5	83.3%
IA	2.4	2.5	0.1	4.2%
MA	1.4	1.8	0.4	28.6%
MD	4.9	4	-0.9	-18.4%
ME	1.9	3.2	1.3	68.4%
MI	1.6	1.8	0.2	12.5%
MN	2.5	2	-0.5	-20.0%
NC	2	2.3	0.3	15.0%
OH	1.8	1.9	0.1	5.6%
OK	3.2	3.3	0.1	3.1%
OR	1.7	3.3	1.6	94.1%
RI	4	4.2	0.2	5.0%
UT	4.9	4.8	-0.1	-2.0%
VA	1.4	1.6	0.2	14.3%
VT	4.1	5.6	1.5	36.6%
Mean	2.92	3.1	0.18	6.2%

Data source: NVDRS 2016 and 2022, for states represented in both survey years and with over 90% of decedents having recorded alcohol involvement measures

To further explore the relationship between alcohol and firearm involvement, Fig. 3 features predicted homicide and suicide deaths by sex, age group and alcohol and firearm involvement derived from the regression results in Table 3. In the upper panel, the predicted number of homicide deaths for males is of higher magnitude in each category than for females, but the overall pattern is similar. Firearms are the predominant means of homicide for both men and women and firearm involvement peaks for those aged 20 to 29. Alcohol combined with firearm involvement followed a similar pattern, peaking at the 20-to-29-year age group and declining subsequently with age. The pattern for suicide (lower panel) is quite different, where firearm use increases slightly for the 20-to-29-year age group, remains relatively stable until the 50-to-59-year age group, and then increases significantly for those age 60 and above. Alcohol use alone and in combination with firearms increased for the 20-to-29-year age group and then remains relatively steady with increasing age. Alcohol alone and along with firearm involvement follows a similar pattern with predicted deaths for women similar to those of men but of a much lower magnitude.

## Discussion

Our results, based on 204,739 decedents from 24 states, show that 26.6% of violent deaths featured alcohol involvement. We also find that alcohol was significantly associated with a higher prevalence of firearm-related suicide deaths, but a lower prevalence of firearm-related homicide deaths, which may reflect the fact that the NVDRS only contains data on alcohol involvement among decedents. These relationships varied by both geographic and demographic characteristics.

Our findings reveal substantial interstate variation in alcohol-involved mortality rates. For instance, North Dakota’s alcohol-involved mortality at nearly 8 per 100,000 was the highest observed, primarily driven by one of the nation’s highest suicide rates in 2022 at 22.5/100,000. In contrast, Oregon’s alcohol-involved death rate is closer to the 24-state average of 3.2, but had the highest observed contribution of alcohol-involved homicide (40%) and, among the sample of states with data over multiple years, experienced the largest percent increase (94%) in alcohol-related violent deaths between 2016 and 2022. Maryland’s above-average alcohol-related mortality rate stems primarily from other (non-homicide and non-suicide) manners of death, some of which may include polysubstance (e.g., opioid-alcohol) use [28]. These observations highlight how state-specific mortality patterns can reflect differences in coding of violent deaths as well as different underlying conditions driving homicide, suicide, and alcohol use among states.

Maryland aside, suicide deaths constituted the largest share of alcohol-involved mortality rates in all other states. Our findings suggest that alcohol plays a pronounced role in suicides, particularly among younger decedents, which is consistent with evidence that alcohol use may amplify behavior leading to firearm use for self-harm at higher rates than in homicide cases [22, 29].

Past research links alcohol to intimate partner violence, and aggression among homicide offenders [7, 8, 23, 30, 31]. Our findings relating to lower alcohol-involvement among homicide decedents are largely explained by the fact that the NVDRS contains only decedents, not offenders. Notably, these decedents, as the victims of homicides, may not be the ones exhibiting the aggressive behaviors associated with alcohol use [32]. This distinction is critical when interpreting our results, as the relationship between alcohol and violent death likely manifests differently depending on whether one examines perpetrators or victims.

This study examines a wide population of decedents to identify the contribution of alcohol to violent deaths rather than a narrow focus on any subpopulation. Compared to past studies, results presented here reveal patterns that might otherwise remain obscured by focusing on only a subset of decedents or a specific state [14, 22, 23, 28, 33–35]. Our estimates of alcohol involvement are also largely in line with findings from the previous century, suggesting that alcohol involvement in violent deaths has been relatively consistent over time [36]. We note, however, that these findings have yet to be validated with a full sample of all US states over multiple years due to a lack of available data.

This study also identified an important intersection of alcohol-involvement with firearms. Scholarship suggests that states with more restrictive alcohol policy environments may see reductions in homicide rates [37, 38], and other studies suggest that the density of alcohol stores [39] and even alcohol advertising [40] may have effects on violent crime. Given that states have broad authority to regulate access, sales, licensing and ownership of both alcohol and firearms, these findings suggest that future research should hone in on these important policy intersections to determine which policy combinations may be most effective.

Our findings advance the understanding of alcohol-firearm mortality relationships in three ways. First, our composite measure of alcohol involvement addresses inconsistencies in alcohol testing and suspicion data across states and time periods. Second, we reveal how state alcohol-involved mortality rates (e.g., North Dakota, Oregon, and Maryland) may emerge from fundamentally different pathways, highlighting the need to further investigate factors driving these distinct risk profiles across different state contexts, including both administrative factors (differences in alcohol testing across states) as well as substantial health policy and population-based differences. Third, the state-specific patterns we identify, particularly the divergence in alcohol’s association with suicide versus homicide deaths, establish a foundation for future evaluations of interventions intended to reduce the role of alcohol on violent deaths.

### Limitations

This descriptive study has several important limitations. The NVDRS does not capture non-fatal injuries, so our results do not reflect the full picture of how alcohol may contribute to violent harms. Not all states participate in the NVDRS, so our results, while valid for included states, cannot be extrapolated to non-participating ones. Testing for alcohol involvement varies within states. To address this limitation, we restricted our samples to states and years where reporting on alcohol involvement was largely complete, but there is still the possibility for misclassification bias. Due to our decision to rely on toxicology testing whenever available, it is likely that the estimates presented here are conservative. Finally, although this analysis explored spatial, temporal, and socio-demographic characteristics associated with different combinations of alcohol and firearms, it does not consider the contribution of other substances or weapons in describing patterns of violent death and does not assess the contribution of policy or other environmental factors.

## Conclusions

Alcohol is present in a significant share of decedents of violent deaths in the United States, and this is the case even when firearms are not used. Interventions aimed at reducing violent deaths may benefit from a greater focus on alcohol. Greater state participation and improved alcohol reporting in the NVDRS would strengthen our ability to monitor and inform such interventions.

## Supplementary Information

Supplementary Material 1

## Data Availability

This research uses data from NVDRS-RAD, a surveillance system designed by the Centers for Disease Control and Prevention’s (CDC) National Center for Injury Prevention and Control. The findings are based, in part, on the contributions of the funded states/territories/jurisdictions that collected violent death data and the contributions of their partners, including personnel from law enforcement, vital records, medical examiners/coroners, and crime laboratories. The analyses, results, and conclusions presented here represent those of the authors and do not necessarily reflect those of CDC. Persons interested in obtaining data files from NVDRS should contact CDC’s National Center for Injury Prevention and Control, 4770 Buford Hwy, NE, MS 106-10, Atlanta, GA 30341-3717, (800) CDC-INFO (232-4636).
