# Supplementary material for: Alcohol and violent deaths in the United States, 2015–2022
Source: Inj Epidemiol. 2026 Mar 26;13:31. doi: 10.1186/s40621-026-00675-4 (PMC13141262; doi:10.1186/s40621-026-00675-4)
Supplement: Supplementary file 1 — Supplementary Material 1 [file 40621_2026_675_MOESM1_ESM.docx]

**ALCOHOL AND VIOLENT DEATHS IN THE UNITED STATES, 2015-2022**

**APPENDICES**

**Appendix Figure 1: Year-to-Year Changes in Alcohol Involvement Metrics from 2016 to 2022 for Selected States**

Note: This figure plots trends in alcohol involvement metrics over time in each state, showing the proportion of total deaths with alcohol testing performed (blue line), or alcohol suspicion status recorded (green lines). We ran year-to-year binomial regressions for each state to identify whether changes in either metric are statistically significant. Statistically significant changes from the prior year to the current year are plotted as triangles.

**Appendix Figure 2: Number of Decedents by Alcohol Status Over Time for Selected NVDRS Population, 2015-2022**

Note: This figure displays the number of decedents over time in the selected NVDRS population from 24 states from 2015 to 2022 categorized by alcohol status. The stacked areas represent distinct but not necessarily exhaustive groups: (1) decedents with recorded alcohol testing results (green), (2) decedents with recorded alcohol suspicion (red), and (3) the total number of decedents in the dataset (blue). The increase in all categories over time reflects both a growth in state participation in the NVDRS and potentially greater documentation of alcohol involvement in participating states. The layering of categories indicates overlap, meaning that some decedents with alcohol testing results are included in the suspicion category.

**Appendix Figure 3: Performance of Varying Firearm Involvement Measures Suggests Undercounting by Relying on ICD-10 Codes, Overcounting by Relying on Narrative String Searches, and Strong Performance by NVDRS Staff-coded Weapon Type variable(s)**

Note: Figure compares the performance of different measures for identifying firearm involvement in deaths within the NVDRS from 2003 to 2022. The results suggest that reliance on ICD-10 codes (green and red lines) tends to undercount firearm-related deaths, while methods based on narrative text searches (purple line) may overcount due to broader inclusion criteria. The NVDRS staff-coded WeaponType variable (blue and dark blue lines) appears to provide the most consistent and reliable classification.

**Appendix Figure 4: Percentage of All Decedents with one or more Alcohol Status Measures Recorded by State, 2015-2022**

Note: States with above 90% of deaths having at least one of the two alcohol involvement measures (testing results or suspicion) coded (or not missing) are kept for our analyses, and outlined in black and labeled. States failing to meet this threshold or who did not participate in the NVDRS are excluded (and outlined in grey, shaded with corresponding recording status). States are not to scale.
